# Expression of Multidrug Resistance-Associated Protein 2 in Human Gallbladder Carcinoma

**DOI:** 10.1155/2013/527534

**Published:** 2013-06-16

**Authors:** Hyun-Soo Kim, Nam Chul Kim, Kyu Hee Chae, Gun Kim, Won Seo Park, Yong-Koo Park, Youn Wha Kim

**Affiliations:** ^1^Department of Experimental Analysis, Aerospace Medical Center, Republic of Korea Air Force, P.O. Box 335-21, 635 Danjae-ro, Namil-myeon, Cheongwon-gun, Chungcheongbuk-do 363-849, Republic of Korea; ^2^Department of Pathology, Graduate School of Medicine, Kyung Hee University, 26 Kyunghee-daero, Dongdaemun-gu, Seoul 130-701, Republic of Korea; ^3^Department of Surgery, Graduate School of Medicine, Kyung Hee University, 26 Kyunghee-daero, Dongdaemun-gu, Seoul 130-701, Republic of Korea

## Abstract

Gallbladder carcinoma (GBCA) is one of the most aggressive malignancies. It is usually diagnosed at an advanced stage, and prognosis remains poor despite advances in imaging techniques and aggressive surgical treatment. Overexpression of multidrug resistance-associated proteins (MRPs) in tumor cells is a major cause of the intrinsic multidrug resistance phenotype. Despite the documented importance of MRP expression in many carcinomas, the prognostic significance of MRP2 expression in primary GBCA is not known. Immunostaining for MRP2 was performed on tissue samples obtained from 143 patients with GBCA. We examined the association between MRP expression and clinicopathological characteristics and outcome of patients with GBCA. GBCA demonstrated MRP2 immunoreactivity in the apicolateral membranes of epithelial cells. MRP2 expression was positive in 53.1% (76/143) of GBCA samples. Positive MRP2 expression was significantly associated with the presence of local recurrence (*P* = 0.038), lymphatic invasion (*P* = 0.038), vascular invasion (*P* = 0.023), and perineural invasion (*P* = 0.006). In addition, the median survival time of patients with MRP2-positive GBCA (15 months) was significantly shorter than that of patients with MRP2-negative GBCA (85 months, *P* = 0.011). We found that the expression of MRP2 in GBCA contributed to aggressive tumor behavior and poor prognosis, suggesting that MRP2 expression can be used as a potential prognostic biomarker of GBCA.

## 1. Introduction

About 0.6% of all patients with cancer in the United States have gallbladder carcinoma (GBCA) or other types of biliary tract carcinoma [1]. In Korea, the incidence of biliary tract carcinomas is 2.5% [2]. The reason for the high incidence of these tumors in Korea is unknown, but it is likely that they are strongly associated with an increased incidence of pigmented stones in the gallbladder and bile ducts. Furthermore, the delayed onset of symptoms and rapid growth of biliary tract carcinomas have resulted in limited therapeutic efficacy and a high mortality rate. Moreover, the role of systemic chemotherapy in palliative treatment of GBCA remains undefined [3]. To date, conventional chemotherapy has been notably ineffective in improving long-term survival of patients with GBCA as these tumors are highly resistant to drug treatment at the onset of therapy. Such chemotherapeutic resistance is a major obstacle to successful cancer treatment [4]. 

ATP-binding cassette (ABC) transporters are a superfamily of membrane proteins that are best known for their ability to transport a wide variety of exogenous and endogenous substances across membranes against a concentration gradient via ATP hydrolysis. The 48 human ABC genes have been classified into seven superfamilies from A to G based on their relative sequence similarities. Subfamily ABC-C includes multidrug resistance-associated protein 1 (MRP1, ABCC1) and the related family members ABCC2 to ABCC9 [4]. MRP1 is widely distributed in normal tissues as well as in the liver, although the level of expression of MRP1 of hepatocytes is low [5]. Apical MRP2 (ABCC2) and basolateral MRP3 (ABCC3) are homologues of MRP1 and play a role in hepatobiliary excretion of bile acids and nonbile acid organic anions [6].

In particular, MRP2 transports a diverse set of substrates and endogenous molecules, such as amphipathic chemicals, drug conjugates, leukotriene C4, prostaglandin, and bilirubin glucuronide and is an important determinant of tissue distribution and elimination [6–8]. The expression and function of this export pump are highly significant in the canalicular membrane of hepatocytes, although other tissues such as the renal proximal tubular cells and intestinal epithelial cells also express MRP2 [9, 10]. MRP2 expression is responsive to a number of drug treatments and is associated with diseases affecting the liver, particularly cholestatic liver disease. Rau et al. [11] found expression of MRP2 in normal human cholangiocytes, suggesting a physiological role of these conjugate export pumps in the secretion of xenobiotics and endogenous anionic conjugates from gallbladder epithelia into blood and bile. 

Overexpression of MRPs in tumor cells is a major cause of intrinsic multidrug resistance phenotype *in vitro* and *in vivo* [11]. MRP2 has been shown to be expressed in lung, gastric, renal, and colorectal carcinoma cell lines [12]. Increased MRP2 mRNA levels have been reported in some cisplain- and doxorubicin-resistant carcinoma cell lines [13, 14]. MRP2 is also expressed in some solid tumors of the kidney, colon, breast, lung, and ovary, as well as in cells from patients with acute myelogenous leukemia [15, 16]. Recently, Korita et al. [17] reported that MRP2 expression determines the efficacy of cisplatin-based chemotherapy in patients with hepatocellular carcinoma.

Despite its documented importance in other carcinomas, there is no report on the prognostic significance of MRP2 in GBCA. In this study, we sought to evaluate the expression of MRP2 in GBCA. We then investigated their association with clinicopathological characteristics and outcomes in patients with GBCA. 

## 2. Materials and Methods

### 2.1. Patients and Tissue Samples

This study included 143 patients with primary GBCA who had not undergone any preoperative chemotherapy or radiotherapy. All patients underwent surgical treatment, as follows: open cholecystectomy with lymph node dissection and concomitant hepatic segmentectomy in 77 patients; laparoscopic cholecystectomy with lymph node dissection in 17 patients; open cholecystectomy with concomitant hepatic segmentectomy in 28 patients; and laparoscopic cholecystectomy alone in 21 patients. 

We reviewed all hematoxylin and eosin-stained slides and performed imunohistochemical staining on the most representative slide from each case. Clinicopathological characteristics, including sex, age, tumor size, histological grade, pathological tumor (T) stage, nodal and distant metastases, TNM stage, local recurrence, lymphovascular invasion, perineural invasion, and resection margin status, were assessed. The tumors were postoperatively staged according to the American Joint Committee on Cancer staging system [18]. No distant metastasis was identified at the time of surgery. Informed consent was obtained from all participants.

### 2.2. Immunohistochemical Staining and Assessment

MRP2 expression was assessed by immunohistochemistry using the Bond Polymer Intense Detection System (Vision BioSystems, Mount Waverley, VIC, Australia) according to the manufacturer's instructions. Briefly, 4 *μ*m sections of formalin-fixed, paraffin-embedded tissue were deparaffinized with Bond Dewax Solution (Vision BioSystems), and an antigen retrieval procedure was performed using Bond ER Solution (Vision BioSystems) for 30 minutes at 100°C. Endogenous peroxidases were quenched by incubation with hydrogen peroxide for 5 minutes. The sections were incubated for 15 minutes at ambient temperature with a mouse monoclonal anti-MRP2 antibody (1 : 100, clone M2 III-6, Abcam, Cambridge, MA, USA). The biotin-free polymeric horseradish peroxidase-linker antibody conjugate system was used in the Bond-maX automatic slide stainer (Vision BioSystems), and antibody-binding was visualized by staining with 3,3-diaminobenzidine (DAB) solution. Nuclei were counterstained with hematoxylin. Slides were dehydrated by a standard procedure and sealed with coverslips. To minimize interassay variation, positive and negative control samples were included in each run. The positive control sample was normal liver. The negative control was prepared by substituting nonimmune serum for primary antibody, which produced no detectable staining.

Immunohistochemical staining of MRP2 was scored semiquantitatively. Briefly, the score was the sum of the percentage of positive tumor cells (0, none; 1, <25%; 2, 25%–49%; and 3, ≥50%) and the staining intensity (0, negative; 1, weak; 2, moderate; and 3, strong). The selection of a cutoff score for positive immunohistochemical expression of MRP2 was based on receiver operating characteristic (ROC) curve analysis. At each score, the sensitivity and specificity of MRP2 expression for the outcome were plotted, thus generating a ROC curve. The score having the closest distance to the point with both maximum sensitivity and specificity was selected as the cutoff score resulting in the greatest number of tumors being correctly classified as having or not having the clinical outcome. In this study, a score of 3 (area under the curve, 0.610; 95% confidence interval (CI), 0.514–0.706; sensitivity, 63.4%; specificity, 60.1%) was determined as the cutoff score for separating the MRP2-positive group (sums between 3 and 6) from the MRP2-negative group (sums between 0 and 2). Two independent pathologists who were blinded to clinicopathological data and patient identity examined and scored all slides independently. Scoring disagreements were resolved by consensus.

### 2.3. Statistical Analysis

The chi-square test or Fisher's exact test was performed to determine whether MRP2 expression in GBCA was associated with the clinicopathological characteristics. For univariate survival analysis, survival curves were estimated using the Kaplan-Meier method, and the log-rank test was used to compute differences between the curves. Multivariate survival analysis was performed on parameters that achieved statistical significance in univariate survival analysis, using the Cox proportional hazards regression model (95% CI) with a backward stepwise elimination method. Differences were considered statistically significant when the *P* value was less than 0.05. SPSS version 15.0 software for Windows (SPSS Inc., IL, USA) was used for statistical analysis.

## 3. Results

### 3.1. Immunohistochemical Expression of MRP2 in GBCA and Its Association with Clinicopathological Characteristics

**Figure 1:**
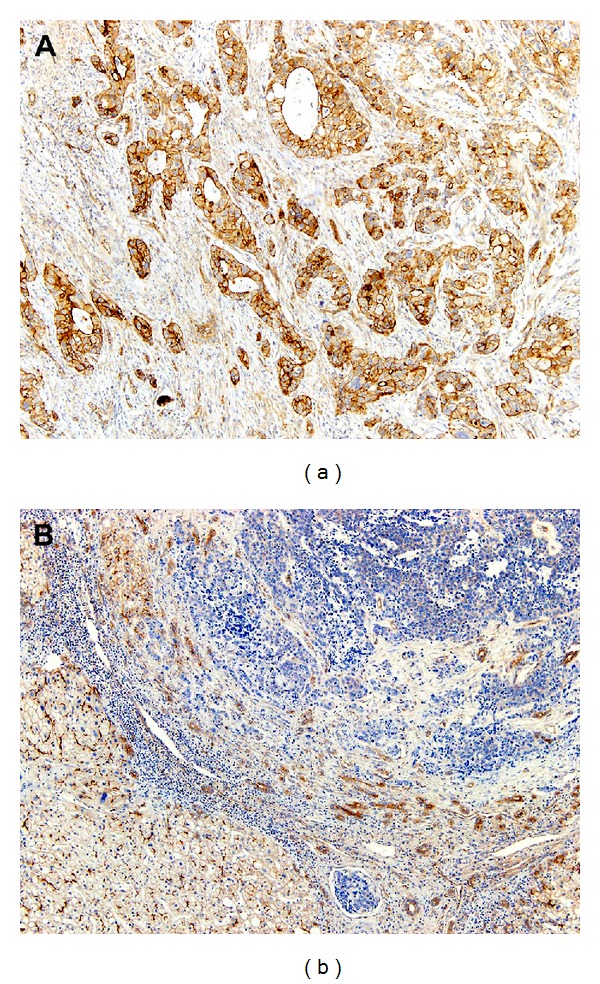
(a) In GBCA, MRP2 immunostaining displayed an apicolateral membranous expression pattern. (b) The absence of MRP2 immunoreactivity in GBCA (right upper corner) contrasted with strong canalicular MRP2 expression in the adjacent hepatocytes (left lower corner). (Polymer method. Original magnification, (a, b), ×100).

In normal hepatocytes, MRP2 showed a canalicular staining pattern. Normal gallbladder mucosa demonstrated immunoreactivity for MRP2 in the apical membrane. MRP2 immunostaining in GBCA showed strong apicolateral membranous expression. Of 143 patients with GBCA, MRP2 expression was positive in 76 (53.1%; Figure 1(a)) and negative in 67 (46.9%; Figure 1(b)). In correlating MRP2 expression with clinicopathological parameters (Table 1), positive MRP2 expression was significantly associated with the presence of local recurrence (*P* = 0.038), lymphatic invasion (*P* = 0.038), vascular invasion (*P* = 0.023), and perineural invasion (*P* = 0.006).

### 3.2. Influence of MRP2 Expression on Overall Survival

**Figure 2:**
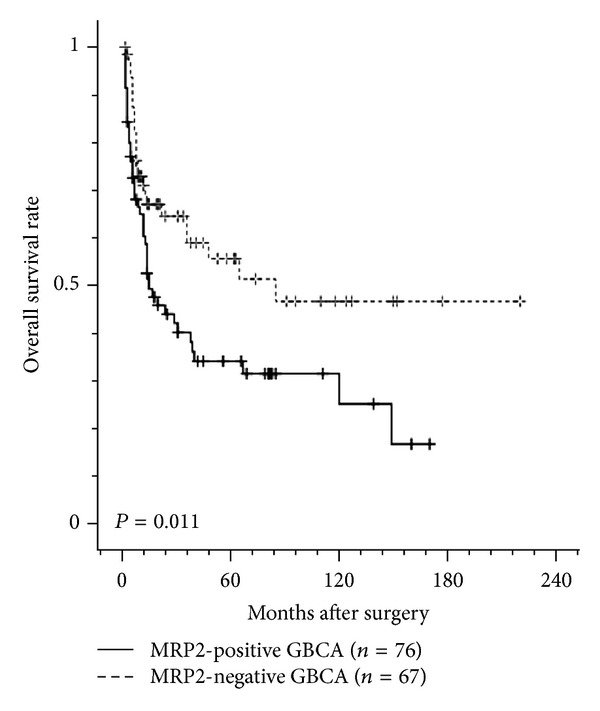
Kaplan-Meier survival curve for overall survival according to the status of MRP2 expression in 143 patients with GBCA. The median survival of patients with MRP2-positive GBCA (15 months) was markedly shorter than that of patients with MRP2-negative GBCA (85 months; *P* = 0.011).

We investigated the prognostic value of immunohistochemical expression of MRP2 in GBCA (Table 2). Adequate clinical follow-up information was available for 143 patients. Of the 143 patients, 78 (54.5%) died during the follow-up period and the remaining 65 (45.5%) were alive at the end of the study. The survival curves according to MRP2 expression status are shown in Figure 2. The median survival of patients with MRP2-positive GBCA (15 months) was markedly shorter than that of patients with MRP2-negative GBCA (85 months; *P* = 0.011). The 1-year, 3-year, 5-year, and overall survival rates were 60.3%, 38.2%, 31.6%, and 16.8%, respectively, for patients with MRP2-positive GBCA and 71.0%, 58.9%, 51.3%, and 46.7%, respectively, for patients with MRP2-negative GBCA.

To estimate the clinical significance of various prognostic factors that might influence survival, univariate survival analyses were performed. Higher histological grade (*P* < 0.001), advanced pathological T stage (*P* < 0.001), the presence of nodal (*P* = 0.034) and distant (*P* = 0.028) metastases, advanced TNM stage (*P* < 0.001), local recurrence (*P* < 0.001), the presence of lymphatic (*P* < 0.001) and vascular (*P* = 0.005) invasions, perineural invasion (*P* = 0.002), tumor involvement of resection margin (*P* = 0.013), and positive MRP2 expression (*P* = 0.011) were significant risk factors affecting overall survival of patients with GBCA (Table 2). To determine the independent prognostic impacts of these factors, multivariate survival analyses were performed using the Cox proportional hazards model. Histological grade (hazard ratio (HR), 2.074; 95% CI, 1.196–3.596; *P* = 0.009), pathological T stage (HR, 2.277; 95% CI, 1.279–4.054; *P* = 0.005), local recurrence (HR, 4.595; 95% CI, 2.541–8.309; *P* < 0.001), and resection margin involvement (HR, 2.060; 95% CI, 1.026–4.133; *P* = 0.042) were identified as independent prognostic factors predicting overall survival. MRP2 expression by itself did not predict outcome.

## 4. Discussion

GBCA is a lethal malignancy that is difficult to cure by current treatment. Considering the lack of treatment options for GBCA, there is an urgent need to develop novel therapeutic strategies and to understand the mechanism of drug resistance in order to develop more effective treatment options in the future. Thus, identification of prognostic biomarkers to predict the outcome of patients with GBCA as a therapeutic target is urgently needed.

It has been demonstrated that patients whose tumors exhibited positive levels of MRP2 expression showed worse prognosis than patients with MRP2-negative tumors in many different types of malignancy, including hepatocellular carcinoma, ovarian carcinoma, esophageal squamous cell carcinoma, renal cell carcinoma, nonsmall cell lung carcinoma and pancreatic ductal carcinoma [19–25]. However, regarding biliary tract carcinomas, to date only one investigation has examined the expression of MRPs in GBCA. Rau et al. [11] investigated the immunohistochemical expression of MRP2 in 14 cases of GBCA. Comparing our findings with those of the previous study, the patterns of MRP2 expression in normal hepatocytes (canalicular staining pattern) and normal gallbladder mucosa (apical staining pattern) were identical to each other. In contrast, we could not compare the pattern of MRP2 expression we observed in GBCA with that of the previous study because Rau et al. [11] did not present any photomicrograph of MRP2 expression in the cancerous tissues. The previous study demonstrated that MRP2 was weakly expressed in only 28.6% (4/14) of GBCAs, suggesting that MRP2 does not play a major role in the multidrug resistance phenotype of GBCAs. The frequency of positive MRP2 expression we observed was quite different from that of the previous study. Since a very small number of samples were examined in the previous study, it was difficult to estimate the accuracy of their results and to assess the clinical value of MRP expression. We speculate that the following reasons might also underlie such a discrepancy: differences in intrinsic tumor heterogeneity, antibodies used, use of staining procedures with varying degrees of sensitivity and lack of a standard evaluation method for immunohistochemical staining.

To the best of our knowledge, this is the first study to analyze the immunohistochemical expression of MRP2 and to examine its prognostic significance in a large number of GBCAs. We discovered that positive MRP2 expression in GBCA was significantly associated with the presence of lymphovascular and perineural invasions, as well as local recurrence, suggesting that the aggressive tumor behavior in GBCA could be partially attributed to MRP2. Moreover, the differences in overall survival rate and median survival time between MRP2-positive GBCA and MRP2-negative GBCA were statistically significant, indicating that MRP2 expression was a significant risk factor affecting overall survival. These findings suggested that MRP2 immunostaining provided clinically useful information in GBCA and that MRP2 expression could serve as a useful predictive biomarker for invasiveness and recurrence in GBCA. However, multivariate analysis showed that MRP2 expression by itself did not predict survival. Hence, the poor prognosis for patients with positive MRP2 expression may reflect dependence of MRP2 expression on factors that independently predict outcome, notably higher histological grade, and advanced pathological T stage and local recurrence.

In conclusion, we demonstrated that MRP2 expression was associated with aggressive tumor behavior and predicted shortened overall survival. Changes in MRP2 regulation may potentially promote lymphovascular and perineural invasions in GBCA, and the level of MRP2 expression may serve as a useful biomarker for local recurrence and patient outcome.

## Figures and Tables

**Table 1:** Relationship between the expression of MRP2 in GBCA and clinicopathological characteristics.

Characteristics	Total	MRP2 expression		
		Positive (%)	Negative (%)	*P* value
Age (year-old)				
≥64	71	42 (59.2)	29 (40.8)	0.153
<64	72	34 (47.2)	38 (52.8)	
Sex				
Man	70	38 (54.3)	32 (45.7)	0.789
Woman	73	38 (52.1)	35 (47.9)	
Histologic grade				
1 (well differentiated)	56	29 (51.8)	27 (48.2)	
2 (moderately differentiated)	66	38 (57.6)	28 (42.4)	0.736
3 (poorly differentiated)	21	9 (42.9)	12 (57.1)	
Tumor size (cm)				
≥2.5	72	38 (52.8)	34 (47.2)	0.929
<2.5	71	38 (53.5)	33 (46.5)	
Pathological T stage				
pT1	23	9 (39.1)	14 (60.9)	
pT2	80	47 (58.8)	33 (41.3)	0.743
pT3	28	14 (50.0)	14 (50.0)	
pT4	12	6 (50.0)	6 (50.0)	
Nodal metastasis				
Present	50	26 (52.0)	24 (48.0)	
Absent	83	47 (56.6)	36 (43.4)	0.604
Unknown	10			
Distant metastasis				
Present	25	13 (52.0)	12 (48.0)	0.899
Absent	118	63 (53.4)	55 (46.6)	
TNM Stage				
I	17	6 (35.3)	11 (64.7)	
II	43	29 (52.4)	14 (32.6)	
III	63	33 (52.4)	30 (47.6)	0.868
IV	10	5 (50.0)	5 (50.0)	
Unknown	10			
Local recurrence				
Present	25	18 (72.0)	7 (28.0)	0.038*
Absent	118	58 (49.2)	60 (50.8)	
Lymphatic invasion				
Present	60	38 (63.3)	22 (36.7)	0.038*
Absent	83	38 (45.8)	45 (54.2)	
Vascular invasion				
Present	36	25 (69.4)	11 (30.6)	0.023*
Absent	107	51 (47.7)	56 (52.3)	
Perineural invasion				
Present	29	22 (75.9)	7 (24.1)	0.006*
Absent	114	54 (47.4)	60 (52.6)	
Resection margin involvement				
Present	17	10 (58.8)	7 (41.2)	0.617
Absent	126	66 (52.4)	60 (47.6)	

*Statistically significant.

**Table 2:** Factors predicting worse outcome of patients with GBCA (univariate and multivariate survival analyses).

Characteristics	Univariate	Multivariate	
	P value	HR (95% CI)	P value
Age (years old), ≥64 versus <64	0.920	Not applicable	
Sex, man versus woman	0.067	1.582 (0.706–2.257)	0.187
Histological grade, 2/3 versus 1	0.001*	2.074 (1.196–3.596)	0.009*
Tumor size (cm), ≥2.5 versus <2.5	0.500	Not applicable	
Pathological T stage, pT3/4 versus pT1/2	<0.001*	2.277 (1.279–4.054)	0.005*
Nodal metastasis, present versus absent	0.034*	0.740 (0.333–1.643)	0.460
Distant metastasis, present versus absent	0.028*	1.353 (0.732–2.500)	0.335
TNM stage, III/IV versus I/II	<0.001*	1.693 (0.912–3.140)	0.095
Local recurrence, present versus absent	<0.001*	4.595 (2.541–8.309)	<0.001*
Lymphatic invasion, present versus absent	<0.001*	1.063 (0.524–2.159)	0.866
Vascular invasion, present versus absent	0.005*	1.092 (0.620–1.924)	0.760
Perineural invasion, present versus absent	0.002*	1.162 (0.601–2.249)	0.655
Resection margin involvement, present versus absent	<0.013*	2.060 (1.026–4.133)	0.042*
MRP2 expression, positive versus negative	0.011*	1.473 (0.877–2.476)	0.143

*Statistically significant.
